# Methodological lacunae in recruitment of HIV positive persons in randomized controlled trials

**DOI:** 10.1186/1471-2334-14-S3-P10

**Published:** 2014-05-27

**Authors:** Arun K Sharma, Vikas K Meena, OP Rajoura, Kuldeep Kumar

**Affiliations:** 1 Department of Community Medicine, University College of Medical Sciences, Delhi-110095, India; 2 Department of Medicine, University College of Medical Sciences, Delhi-110095, India

## Background

Recruitment of patients in an RCT is crucial to the success of the trial. The sample selection and sample size estimation is based on previously reported research studies. We conducted an RCT to assess the effectiveness of mobile text messaging in improving adherence to highly active anti retroviral therapy in an ART unit of a tertiary care hospital. The present study was aimed to examine the impact of recruitment process and sampling on the outcome of the trial.

## Methods

The required sample size for recruitment in the RCT was calculated based on a reported defaulter of 27% in a previous Indian study. In order to attain an effect size of 0.3 for 1 degree of freedom, the required sample size in each arm was 44. Recruited 60 persons in each arm taking into account 20% drop out and 15% mortality rate.

## Results

Analysis of data showed that the initial assumptions were not matching. Loss to follow up due to death and attrition were 4.1% and 6.6% respectively. Using person months of observation, corresponding loss was only 2.6% and 1.2% respectively. Default rates in the intervention and control arms were 1.6% and 2.3% respectively (not significant).

## Conclusion

The default rates were much lower than the a priori hypothesized values. The study suggests that selection of cases based on data from a different center may result in wrong estimation of sample size. ART defaulter is more likely to be a function of factors which are not influenced by mobile text messaging.

